# CEG 2.0: an updated database of clusters of essential genes including eukaryotic organisms

**DOI:** 10.1093/database/baaa112

**Published:** 2020-12-11

**Authors:** Shuo Liu, Shu-Xuan Wang, Wei Liu, Chen Wang, Fa-Zhan Zhang, Yuan-Nong Ye, Candy-S Wu, Wen-Xin Zheng, Nini Rao, Feng-Biao Guo

**Affiliations:** School of Life Science and Technology, Center for Informational Biology, University of Electronic Science and Technology of China, Chengdu 610054, China; Key Laboratory of Combinatorial Biosynthesis and Drug Discovery, Ministry of Education and School of Pharmaceutical Sciences, Wuhan University, Wuhan 430071, China; School of Life Science and Technology, Center for Informational Biology, University of Electronic Science and Technology of China, Chengdu 610054, China; School of Life Science and Technology, Center for Informational Biology, University of Electronic Science and Technology of China, Chengdu 610054, China; School of Life Science and Technology, Center for Informational Biology, University of Electronic Science and Technology of China, Chengdu 610054, China; School of Life Science and Technology, Center for Informational Biology, University of Electronic Science and Technology of China, Chengdu 610054, China; Bioinformatics and BioMedical Bigdata Mining Laboratory, Key Laboratory of Environmental Pollution Monitoring and Disease Control, Ministry of Education, Guizhou Medical University, Guiyang 550025, China; Thomas Worthington High School, 300 West Granville Road, Worthington, OH 43085, USA; School of Biomedical Engineering, Capital Medical University, Beijing 100069, China; School of Life Science and Technology, Center for Informational Biology, University of Electronic Science and Technology of China, Chengdu 610054, China; Key Laboratory of Combinatorial Biosynthesis and Drug Discovery, Ministry of Education and School of Pharmaceutical Sciences, Wuhan University, Wuhan 430071, China

## Abstract

Essential genes are key elements for organisms to maintain their living. Building databases that store essential genes in the form of homologous clusters, rather than storing them as a singleton, can provide more enlightening information such as the general essentiality of homologous genes in multiple organisms. In 2013, the first database to store prokaryotic essential genes in clusters, CEG (Clusters of Essential Genes), was constructed. Afterward, the amount of available data for essential genes increased by a factor >3 since the last revision. Herein, we updated CEG to version 2, including more prokaryotic essential genes (from 16 gene datasets to 29 gene datasets) and newly added eukaryotic essential genes (nine species), specifically the human essential genes of 12 cancer cell lines. For prokaryotes, information associated with drug targets, such as protein structure, ligand–protein interaction, virulence factor and matched drugs, is also provided. Finally, we provided the service of essential gene prediction for both prokaryotes and eukaryotes. We hope our updated database will benefit more researchers in drug targets and evolutionary genomics.

**Database URL:** http://cefg.uestc.cn/ceg

## Introduction

Essential genes are types of irreplaceable gene sets (1). Their deletion or mutation can cause fitness decrease. These core elements can be used for minimal genome synthesis (2) and are the potential targets of drug design. Several databases have been constructed to collect and organize essential genes, among which DEG (Database of Essential Genes) (3) and OGEE (database of Online GEne Essentiality) (4) are the most well-known ones. However, before 2013, there were no databases to reserve essential genes in functional homologous clusters. In 2013, we constructed CEG (5), a database of clusters of essential genes. The entries in CEG are stored and organized into homologous clusters in different species, which allow us to infer the conservativeness and specificity of gene essentiality. Some genes have essential homologues in many species, whereas other genes may have essential homologues in only partial species, though homologous genes may still appear in many species. Each of our clusters has one specific CEG ID and belongs to one specific COG (Cluster of Orthologous Groups of proteins) (6). The database also provides references for anti-bacterial drug target selection through the *e*-value of the best blast match between essential gene-encoding proteins and human proteins.

Since the release of CEG version 1, it has been used in the research of drug-target screening, metabolic network analyses and essential gene predictions. For example, Nayak and colleagues used CEG as a reference database to screen potential drug targets for pathogens causing bacterial pneumonia (7). It was also used to determine the essentiality of metabolism-associated enzymes in *Chromohalobacter salexigens* (8). The first release of CEG included a total of 2861 clusters covered by 16 prokaryotic gene datasets.

Seven years have passed since then, and the quantity and quality of essential genes have increased significantly. To provide more comprehensive data and landscape of essential gene clusters, we have updated our CEG database. The current version of CEG includes a total of 4421 prokaryotic clusters and 5936 eukaryotic clusters. Furthermore, to help drug design, we have extracted information such as structure of essential genes, essential gene-targeting drugs and essential gene-interacting ligands from public databases.

## Materials and methods

### Main data storage and cluster assignment

The gene essentiality data in CEG 2.0 were downloaded from DEG (3) and OGEE (4), and a total of 29 prokaryotic gene datasets from 24 species were included. They belonged to 5 different phyla including *Proteobacteria* (18 gene datasets), *Firmicutes* (5 gene datasets), *Tenericutes* (2 gene datasets), *Actinobacteria* (1 gene dataset) and *Bacteroidetes* (3 gene datasets). Among the 24 species, 3 species, including *Staphylococcus aureus, Pseudomonas aeruginosa* and *Salmonella enterica*, each contained more than 1 gene dataset. Newly added data were clustered according to their COG ID. The principle is that genes with the same COG ID will be clustered together, and for those without a specific COG ID, homologous alignments will be used to endow its COG ID according to genes best matched with it. The detailed information comparing the two database versions is listed in Table S1, which includes gene numbers and cluster numbers of prokaryotes, eukaryotes and human. The distribution of numbers of clusters with specific cluster size is provided in Figure S1.

CEG 2.0 also includes eukaryotic essential genes from nine eukaryotes, including *Arabidopsis thaliana, Aspergillus fumigatus, Caenorhabditis elegans, Danio rerio, Drosophila melanogaster, Saccharomyces cerevisiae, Schizosaccharomyces pombe* 972h-, *Mus musculus* and *Homo sapiens*, from DEG 15.2 and OGEE. Genes are then assigned to specific clusters according to OrthoDB V9.1 (9). Using the API (Application Programming Interface) data interface of OrthoDB (version 9.1) and crawler, the genes and their homologous genes were obtained together with a specific EOG ID. If genes or their homologous genes were not clustered into any specific EOG, we made alignments between homologous genes and other essential genes with clear EOG ID to find their possible EOG ID.

For further investigation of essential genes in multiple human cancer cell lines, CEG 2.0 constructs a page to store human essential genes in clusters. Totally 11 cell lines named KBM7, HAP1, K562, Jiyoye, Raji, A375, DLD1, GBM, HCT116, HELA and REP1 and 1 general group named ‘human’ are included. Genes are then assigned to specific clusters according to their EOG ID.

### Collection of drug-related information

Similar to the first version, the new version provides sequence similarity information (*e*-value of blastp) between added essential genes and human protein-coding genes, which helps identify genes that cause the least toxicity as drug targets. The structure information for protein molecules bound to each gene can be accessed through the link provided in the ‘Struct’ column. The ‘Protein-Ligand’ column provides all of the possible binding forms for the essential protein and the ligand to bind in BioLip (https://zhanglab.ccmb.med.umich.edu/BioLiP/qsearch.html) (10). Some essential genes are potential virulence factors (11) and have been highlighted. More importantly, we listed drug molecules for some of the essential genes with approved or currently testing drugs in DrugBank (12). The abovementioned information could help researchers better understand applicable essential genes, which can be chosen as anti-bacterial drug targets. Their detailed numbers are listed in Table 1.

**Table 1.** Drug-associated information newly updated in CEG 2.0

	Ligand–protein interaction	Protein structure	Virulence factors	Matched drugs
Entry number	9784	3165	909	855
Gene number	552	825	909	2754
Cluster number	453	627	395	439
Gene dataset number	15	15	29	29

In order to show the structure of a prokaryotic cluster, we use CEG0128 as an example. The cluster size and strain size are both 16, suggesting the general importance of this cluster. Among the genes, 16 of them can find glycerin to be the related drug, whose accession number in DrugBank is DB09462 (https://www.drugbank.ca/drugs/DB09462). In regard to virulence factors, 2 out of 16 genes have been confirmed as virulence-related genes, while the other 14 genes are potential virulence factors. One of the 16 genes, marked as CEG0128_16130686, has information on ligand–protein interactions. By clicking on the arrow shown in the corresponding column, users can access a link to the detailed information of those entries. Three PDB IDs for the essential protein (1e9i, 2yfm and 3h8a), two ligand IDs (III and MG) and two binding sites (BS01 and BS02) are contained. There are 5 total essential protein chains (called receptors), A, B, C, D and F; the number of binding site residues is 28 after removing redundancies.

### CEG 2.0 implementation

**Figure 1.**
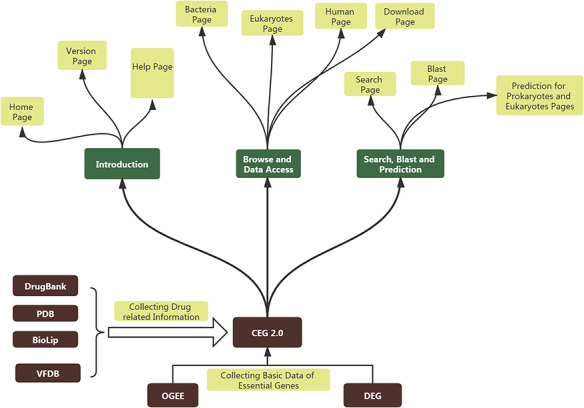
The basic scheme for constructing CEG 2.0 and the structure of CEG 2.0.

CEG 2.0 database stores data in MySQL database and utilizes a Linux system. The interface mainly uses HTML and PHP (hypertext preprocessor) languages. Python language is used to enable users to search using keywords in our database and their blast sequences. CEG 2.0 also provides predictions of essentiality by using items such as gene names and sequences of prokaryotes or eukaryotes, which are mainly implemented in Python. The basic theory for constructing CEG 2.0 and its general sub-pages can be accessed in Figure 1.

## Results and notes for CEG 2.0

### General statistical data for CEG 2.0

CEG 2.0 includes a total of 29 prokaryotic essential gene datasets from 5 phyla, eukaryotic essential genes of 9 eukaryotes and 26 971 essential genes grouped into 5098 clusters of 12 human cancer cell lines. Compared to the first version, the number of prokaryotic essential genes increased from 6738 to 11 884, 13 extra prokaryotic essential gene datasets were added and the cluster number increased from 2861 to 4421. For the 9 new eukaryotes, 12 728 non-redundant essential genes were grouped into 5936 clusters.

### General structure of CEG 2.0

CEG 2.0 includes 11 pages. The ‘Home’ page describes the properties of CEG 2.0, the method used to group genes into clusters and contact information of the webmaster for when users have trouble using CEG 2.0. The ‘Version’ page compares the basic statistics of CEG 2.0 with that of the first version. Information for drugs is highlighted in one table. Three browsing pages specifically designed for essential genes of bacteria, eukaryotes and humans are incorporated. On the ‘Bacteria’ page, users can open a new page to see all members of one cluster, whether a gene has functional drugs (https://www.drugbank.ca/drugs), or whether its encoding protein can be bound to a ligand (https://zhanglab.ccmb.med.umich.edu/BioLiP/). The best-matched score for genes in a cluster is also provided for users to judge whether drugs targeting a gene-encoding protein may cause severe toxicity. Finally, we have provided virulence information (http://www.mgc.ac.cn/VFs/main.htm) and associated pathways (https://www.kegg.jp/) of each gene. The digital information for clusters can be displayed in ascending or descending order of each attribute’s size.

The ‘Eukaryotes’ page and ‘Human’ page provide the cluster information of eukaryote and human cancer cell lines. Users can also sort clusters by cluster size (number of genes in a cluster) or species size (number of species or cancer cell lines in a cluster) in ascending or descending order.

Users can turn to the ‘Search’ page if they would like to find information about a gene or cluster using keywords such as gene name, cluster size, COG ID and EOG ID. The ‘Blast’ page enables users to quickly find sequences that best match their genes. Similarity scores are all provided thereafter.

If users would like to download the original data from CEG 2.0, they can navigate to the ‘Download’ page. The CSV and DAT files are available for access. Here we also provide a standalone software version named CEG_Match_V1.5 for users to predict the essentiality of their gene freely. If a user has any questions about CEG 2.0, they can navigate to the ‘Help’ page for assistance. Furthermore, CEG 2.0 provides services for users to predict genes of prokaryotes or eukaryotes in two separate pages through inputting gene names or sequences into the prediction box.

### Predict the essentiality of genes with gene names or sequences

As mentioned above, CEG 2.0 can predict the essentiality of genes. Such functions are implemented on the ‘Predict’ page for prokaryotes and eukaryotes, respectively. If an investigated gene happens to have essential orthologues in multiple species, the possibility of it being an essential gene increases significantly. Such a possibility increases with the matching species’ number. The information of the matching species is important for prediction. Thus, we estimate the essentiality of input genes based on species size and output the information on matching species for users to make their judgment. Users can adjust the threshold *k* according to their requirements. Those genes with a matching species’ number higher than *k* will be considered as potentially essential.

We will take a gene from *Komagataella phaffii* GS115 as an example. Its UniProt (13) accession is C4QZ40 (https://www.ncbi.nlm.nih.gov/gene/?term=C4QZ40). Note that this species is not present in our current database. According to the information from NCBI, its functional description is a hypothetical protein with nucleotide and amino acid sequence lengths being 1407 nt and 468 aa, respectively. In order to predict its essentiality, we input the protein sequence into the ‘Predict(eukaryotes)’ page of CEG 2.0 and discover that it matches the cluster CEGE0833 in CEG 2.0, containing three species with essential genes. The three species are *H. sapiens, S. cerevisiae* and *S. pombe* 972h. This cluster has the annotated function of mannosyltransferase in other genomes. These genes are experimentally validated as essential genes in *K. phaffii* GS115, and its DEG ID is DEG20270001, reported in 2018 (14). This case can validate the reliability of our prediction result. In fact, we have comprehensively illustrated the reliability of predicting essential genes based on the number of matching species (5).

The CEG_match algorithm is based on annotation of gene function (standard gene names) and sequence alignments between users’ query sequences and sequences stored in our database. We set the minimum hitting number for search by gene names or sequences. Only those genes with hitting number over this threshold are predicted as essential genes. General performance of CEG_match has been tested when reporting the last version of the database (5), where it showed higher accuracy than the direct blast alignment. Later, a third-party review illustrated similar results after strict comparison on a larger-scale benchmark (15). CEG_Match, direct blast alignment (provided by Tubic DEG database) and Geptop (also provided by us) (16), are the only three tools currently available for bacterial essential gene prediction. CEG_match has been reported as less accurate than Geptop, but the latter requires complete proteome sequences as input, while CEG_match could generally give reliable prediction using only one gene (or protein) sequence or gene name as an input. In addition, if the users choose a *k* threshold of 1, then the prediction result of CEG_match will be the same with that of direct blast alignment. Choosing a *k* threshold of 2, 3 or even higher will produce a better balance of sensitivity and specificity, and we leave the choice of different *k* value to the users, only suggesting 3 as the default setting. If users require higher frequency of predicted essential genes to be genuinely essential (i.e. higher precision), then they should use a larger *k* value, or if they want predicted essential genes containing more genuinely essential genes (i.e. higher sensitivity), then they should use a lower *k* value.

### Potential interests within new features of the current database

Users may wonder how such essential genes react together to perform their essential roles, and CEG 2.0 provides this pathway information. The update includes available structure data of essential genes, which can benefit the researchers surrounding research of three-dimensional structures and designing essential gene-targeting drugs. To help design new drugs directly, we extracted related information of ligands and approved or tested drugs targeting essential genes from BioLip and DrugBank databases, for example, the interaction site and three-dimensional structure of their compound. As many essential genes (proteins) have been used as drug target molecules, these essential genes associated structure and ligands would provide insight for drug designing. Take essential gene rpsC (30S ribosomal protein S3) as an example, which belongs to the CEG cluster CEG0007, with a cluster size of 24. According to such a high size-value, it could be a preferred target for anti-bacterial design. In fact, two drugs have been deposited in DrugBank, with IDs DB00759 and DB08185. The former has the chemical formula C_22_H_24_N_2_O_8_, which blocked aminoacyl tRNA from binding to the ribosome acceptor site. The latter is still under experiment, waiting for approval. One item of structures related to this gene is 3J9W. The 3D view of this structure can be accessed from the linked webpage. The binding forms of these essential proteins and ligands are all listed in links of the BioLip database. We think more attention should be paid to these ligands as they may prove to be effective after safety tests.

## Discussion

One gene is essential, but its orthologues in other genomes may or may not be essential genes (17). Generally, this depends on the evolutionary distance of the referred species to the investigated species. However, evolutionary distance is not an absolute measure. Indeed, the actual essentiality of one gene is determined by its genomic circumstances and network system (18). The abovementioned facts constitute the background information for us to construct a database of essential gene clusters. With the cluster database, we can investigate conservation and specificity of one given essential gene and decide its general essentiality in the prokaryotic or eukaryotic domain.

In the current version, we have provided the service of essentiality prediction. Users can choose to input gene names or their nucleotide or protein sequences. In fact, this is a speculation of a gene’s general essentiality. However, this is still more accurate than the direct sequence alignment as it decreases the false-positive rate (5).

General essentiality is calculated on the scale of domain (prokaryotes or eukaryotes). If the cluster size of the hitting gene is quite high (10 or higher in prokaryotes and 3 or higher in eukaryotes), the prediction of being an essential gene will be very reliable. If there is not any essential hitting, then the query would very possibly be non-essential. However, if the hitting cluster size is a medium number, we suggest that users manually curate the prediction results by pondering the evolutionary distance of query sequences’ species and the hitting genes’ species. In this case, only when the query species is closely related to one of the reference species in CEG is the prediction highly reliable. To ensure reliable predictions, we output the species’ names with essential gene hits. With the information of hitting species size and species names, we think the users could easily make their decision of essentiality for their submitted genes.

## Supplementary Material

baaa112_Supp
