# Supplementary material for: CEG 2.0: an updated database of clusters of essential genes including eukaryotic organisms
Source: Database (Oxford). 2020 Dec 11;2020:baaa112. doi: 10.1093/database/baaa112 (PMC7731928; doi:10.1093/database/baaa112)
Supplement: baaa112_Supp [file baaa112_supp.zip › supplementary table1.docx]

**Supplementary table 1.** General statistical data of CEG1.0 and CEG2.0

| Version | Number of prokaryotic gene datasets | Number of eukaryotes | Number of prokaryotic genes | Number of prokaryotic clusters | Number of eukaryotic genes | Number of eukaryotic clusters | Number of human genes | Number of human clusters |
| --- | --- | --- | --- | --- | --- | --- | --- | --- |
| CEG1.0 | 16 | 0 | 6738 | 2861 | 0 | 0 | 0 | 0 |
| CEG2.0 | 29 | 9 | 11884 | 4421 | 12728 | 5936 | 26971 | 5098 |
