# Supplementary figures and images for: CEG 2.0: an updated database of clusters of essential genes including eukaryotic organisms
Source: Database (Oxford). 2020 Dec 11;2020:baaa112. doi: 10.1093/database/baaa112 (PMC7731928; doi:10.1093/database/baaa112)

Distribution of cluster numbers with specific cluster size between CEG1.0 (in red) and CEG2.0 (in blue)

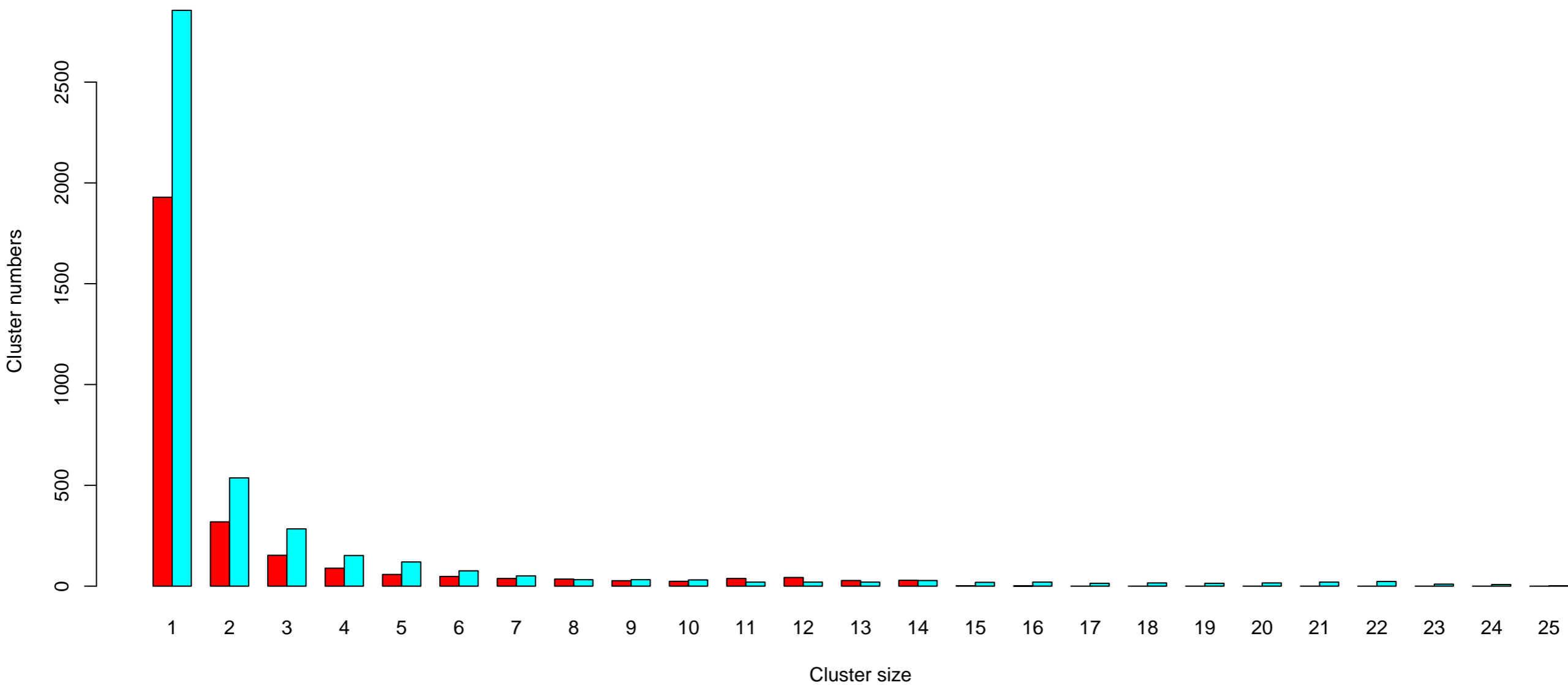

Supplement: baaa112_Supp [file baaa112_supp.zip › supplementary figure1.pdf]
